# Parahydrogen‐Polarized [1‐^13^C]Pyruvate for Reliable and Fast Preclinical Metabolic Magnetic Resonance Imaging

**DOI:** 10.1002/advs.202303441

**Published:** 2023-08-16

**Authors:** Luca Nagel, Martin Gierse, Wolfgang Gottwald, Zumrud Ahmadova, Martin Grashei, Pascal Wolff, Felix Josten, Senay Karaali, Christoph A. Müller, Sebastian Lucas, Jochen Scheuer, Christoph Müller, John Blanchard, Geoffrey J. Topping, Andre Wendlinger, Nadine Setzer, Sandra Sühnel, Jonas Handwerker, Christophoros Vassiliou, Frits H.A. van Heijster, Stephan Knecht, Michael Keim, Franz Schilling, Ilai Schwartz

**Affiliations:** ^1^ Department of Nuclear Medicine, TUM School of Medicine Klinikum rechts der Isar of Technical University of Munich 81675 Munich Germany; ^2^ NVision Imaging Technologies GmbH 89081 Ulm Germany; ^3^ Munich Institute of Biomedical Engineering Technical University of Munich 85748 Garching Germany; ^4^ German Cancer Consortium (DKTK) Partner Site Munich and German Cancer Research Center (DKFZ) Im Neuenheimer Feld 280 69120 Heidelberg Germany

**Keywords:** hyperpolarization, metabolic imaging, magnetic resonance imaging (MRI), parahydrogen, pyruvate

## Abstract

Hyperpolarization techniques increase nuclear spin polarization by more than four orders of magnitude, enabling metabolic MRI. Even though hyperpolarization has shown clear value in clinical studies, the complexity, cost and slowness of current equipment limits its widespread use. Here, a polarization procedure of [1‐^13^C]pyruvate based on parahydrogen‐induced polarization by side‐arm hydrogenation (PHIP‐SAH) in an automated polarizer is demonstrated. It is benchmarked in a study with 48 animals against a commercial dissolution dynamic nuclear polarization (d‐DNP) device. Purified, concentrated (≈70–160 mM) and highly hyperpolarized (≈18%) solutions of pyruvate are obtained at physiological pH for volumes up to 2 mL within 85 s in an automated process. The safety profile, image quality, as well as the quantitative perfusion and lactate‐to‐pyruvate ratios, are equivalent for PHIP and d‐DNP, rendering PHIP a viable alternative to established hyperpolarization techniques.

## Graphical abstract

In this study, the culmination of several recent advances in the polarization of [1‐^13^C]pyruvate via PHIP‐SAH (Parahydrogen Induced Polarization via Side‐Arm Hydrogenation) is presented. In a fully automated process, purified solutions of pyruvate are obtained at physiological pH and preclinically relevant volumes (up to 2 mL), concentrations (70–160 mM), and polarization levels (PHIP: 17.8 ± 1.2%; d‐DNP: 22.8 ± 5.4%) at the time of injection.

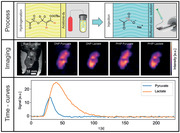

## 1 Introduction

Since its introduction in 1973, magnetic resonance imaging (MRI) has provided non‐invasive insights into living organisms with high soft‐tissue contrast by means of low‐energy radiofrequency fields.^[^ ^1^ ^]^ Imaging physiological functions and microstructure continues to be a major motivation for innovation in the field and has resulted in a plethora of technologies and techniques that have reached clinical applications, including functional MRI (fMRI),^[^ ^2^ ^]^ diffusion‐weighted imaging (DWI),^[^ ^3^ ^]^ and dynamic contrast‐enhanced (DCE)‐MRI.^[^ ^4^ ^]^ A unique capability of magnetic resonance is the ability to assess molecular composition of tissue, using differences in the local magnetic fields experienced by nuclear spins, generating a difference in resonance frequency, also known as chemical shift. This enables liquid‐state NMR spectroscopy techniques that are routinely used in various fields of chemistry, but have not yet been exploited for routine diagnostics in the clinic. This is partly due to the low sensitivity of NMR, resulting from the intrinsic small nuclear spin polarization at thermal equilibrium at clinically achievable field strengths, which prohibits molecular imaging at sufficient resolution. ^1^H‐MR spectroscopy suffers from long acquisition times and crowded spectra due to the relatively small spectral range, an abundance of different ^1^H‐nuclei and strong *J*‐coupling. Conversely, positron emission tomography (PET) offers very high sensitivity, but involves patient exposure to potentially harmful ionizing radiation and cannot directly distinguish different molecules and their downstream metabolites labeled with the same radioactive isotope.

MR's sensitivity issuecan be addressed by hyperpolarization techniques.^[^ ^5^ ^]^ An artificially high nuclear spin polarization, more than four orders of magnitude higher than at thermal equilibrium, is produced in hyperpolarized imaging agents, thereby boosting their measurable signal. In clinical studies, hyperpolarized noble gasses enabled visualization and quantification of lung ventilation^[^ ^6^ ^]^ and hyperpolarized ^13^C‐labeled biomolecules facilitated the real‐time measurement of metabolism in tumors, such as in the prostate^[^ ^7^ ^]^ or in healthy and diseased brains.^[^ ^8^ ^]^

Currently, the most widely used hyperpolarization technique is dissolution dynamic nuclear polarization (d‐DNP), which requires a superconducting magnet and operates at liquid helium temperatures.^[^ ^9^, ^10^, ^11^ ^]^ Although technically demanding, this technique allows a broad range of molecules to be polarized,^[^ ^12^ ^]^ is commercially available and is the standard technique for preclinical and clinical studies. There has been a focus on imaging the metabolism of hyperpolarized [1‐^13^C]pyruvate, as its downstream products allow quantification of both oxidative energy metabolism within the tricarboxylic acid cycle and of glycolytic lactate production.^[^ ^13^ ^]^ Even though its clinical value for diagnosis and treatment response assessment has been demonstrated in several tumor entities, such as in the prostate,^[^ ^7^ ^]^ breast,^[^ ^14^ ^]^ and kidneys,^[^ ^15^ ^]^ the widespread use of hyperpolarized metabolic MRI is currently limited by the high cost and complexity of d‐DNP instruments.^[^ ^11^ ^]^

Novel technologies are needed to lower the hurdle for clinical translation and to allow more widespread use of hyperpolarized MRI. Two promising techniques exploit parahydrogen, a highly‐polarized spin state of hydrogen gas that is accessible at moderate temperatures using liquid nitrogen. Parahydrogen can be used to polarize [1‐^13^C]pyruvate subsequently, via hydrogenation, termed “parahydrogen‐induced polarization (PHIP)” ,^[^ ^16^, ^17^ ^]^ or via reversible exchange with a binding complex, termed “signal amplification by reversible exchange (SABRE)”.^[^ ^18^ ^]^ In cases for which direct hydrogenation is not feasible, such as for pyruvate and lactate, the introduction of the “parahydrogen‐induced polarization by side‐arm hydrogenation (PHIP‐SAH)” approach has increased the applicability of that technique.^[^ ^19^ ^]^

For PHIP‐SAH induced hyperpolarization of pyruvate, the polarization process involves four key steps: 1) Derivatization of the carboxylate group with an unsaturated side‐arm to form a pyruvate ester, 2) parahydrogenation of the unsaturated bond in the side‐arm, 3) transfer of parahydrogen polarization to carbon nuclei and 4) hydrolysis for removal of the side‐arm and catalyst.

Nevertheless, the use of hyperpolarized [1‐^13^C]pyruvate by PHIP‐SAH and SABRE has been severely limited, compared to d‐DNP, due to: 1) insufficient polarization and concentration for the required signal‐to‐noise ratio – so far, in vivo studies were reported with [1‐^13^C]pyruvate polarization levels at the time injection of ≈6%–12% for PHIP‐SAH^[^ ^19^, ^20^ ^]^ and of ≈5%–11% for SABRE^[^ ^21^, ^22^ ^]^, with pyruvate concentration of ≈30–40 mM for both PHIP‐SAH and SABRE; 2) a high impurity profile, including rhodium or iridium from the catalyst and residual solvents that could induce toxicity (e.g., chloroform or methanol). Although the concentration of toxic catalyst solvents in the end product can be reduced – either by phase transfer,^[^ ^19^ ^]^ filtration^[^ ^23^ ^]^ or precipitation and redissolution^[^ ^24^ ^]^ – it still represents a major challenge for the translation to the clinics.

Here, we show the culmination of several novel advances in the polarization of [1‐^13^C]pyruvate via PHIP‐SAH, resulting in a fully‐automated PHIP‐SAH polarizer. Our prototype produces purified, concentrated (≈70–160 mM, depending on application) and highly hyperpolarized (≈18%) solutions of pyruvate at physiological pH and preclinically relevant volumes of up to 2 mL. We benchmark the prototype against a commercial d‐DNP system (HyperSense, Oxford Instruments) in a preclinical study with 48 animals involving rats and mice, either healthy or tumor‐bearing. Newer commercial preclinical^[^ ^25^ ^]^ and clinical^[^ ^26^ ^]^ dissolution DNP systems are also available to date for which reported polarization values of [1‐^13^C]pyruvate at the time of injection exceed 30% polarization and are slightly higher than for the d‐DNP system used in this study.

## 2 Results and Discussion

### 2.1 PHIP Polarizer Setup and Characterization

**Figure 1:**
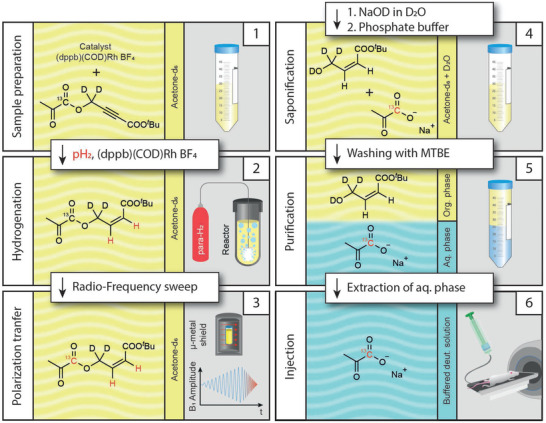
PHIP‐SAH hyperpolarization of [1‐^13^C]pyruvate. First, the pyruvate ester is dissolved, together with the catalyst, in acetone‐d_6_. Second, the ester is hydrogenated with parahydrogen inside a reactor. Third, the polarization is transferred from the hydrogen to the ^13^C nuclei via an rf‐sweep in a magnetic shield. Fourth, the pyruvate is cleaved off and buffered. Fifth, MTBE is added to separate the pyruvate from the organic impurities. Sixth, the drug product is extracted and ready for injection. Note that all six steps are combined in one device (PHIP polarizer, see Figure S1, Supporting Information).

In previous studies, researchers predominantly chose organic solvents for the hydrogenation/polarization process that are poorly soluble in water, allowing a straightforward extraction process after the ester hydrolysis. However, we observed that the hydrogenation and especially the polarization of the ester works best in acetone, which is miscible with water. This poses a challenge for the purification since a simple phase separation and extraction cannot be utilized. We developed a polarization and purification process (**Figure** **1**), which allows for hydrogenation in acetone: Step 1: The ^13^C labeled precursor (pyruvate ester with unsaturated C,C‐triple bond in the side‐arm) and the rhodium catalyst, dissolved in deuterated acetone, are injected into a reaction vessel (see methods section for experimental details). Step 2: The unsaturated triple‐bond of the precursor is then hydrogenated with parahydrogen under elevated pressure and temperature. The side‐arm of the pyruvate ester was tailored for a rapid and highly selective hydrogenation reaction. Step 3: The polarization transfer takes place in a magnetic shield via radio‐frequency sweeps.^[^ ^27^ ^]^ Step 4: Subsequently, the solution is shuttled out of the magnetic shield into a vessel, where it is mixed with sodium hydroxide solution. The addition of the hydroxide initiates the cleavage of the hydrogenated pyruvate ester to sodium pyruvate and the hydrogenated side‐arm. A buffered solution is added to adjust the pH to a physiological value. Step 5: To initiate the phase separation of the organic and the aqueous phase, methyl tert‐butyl ether (MTBE) is added to the mixture. This separates the hydrophilic pyruvate in the aqueous phase from most of the acetone, the hydrogenated side‐arm and catalyst in the organic phase. Thereafter, the aqueous phase is pumped into a vessel containing fresh MTBE as a further washing step, which reduces the organic impurities even further. The side‐arm of the ester is designed for poor water solubility, making the purification process via extraction after cleavage more efficient. Step 6: In the last process step, the aqueous phase is pumped to a heated vessel, in which nitrogen is bubbled through the solution at a reduced pressure. This reduces the MTBE and acetone concentrations to ≈6 and 90 mM, respectively. The whole purification process also reduces the rhodium concentration by more than 95% to under 8 µg mL^−1^ (see **Table** **1**). With these concentrations of catalyst and organic solvents in the injected solutions, we did not observe any adverse health effects even with four injections per rat per hour. The extracted product (≈2 mL) contains 160 mM sodium pyruvate with a polarization in the range of 18%. To investigate lower concentrations, we prefilled the extraction syringe with D_2_O and diluted the high pyruvate concentration coming from the polarizer. The entire process from sample injection to the extraction of the polarized, purified pyruvate takes 85 s. During the whole purification process, the liquid stays inside a 100 mT permanent magnet.

**Table 1:** Impurity concentrations in the extracted pyruvate doses as measured after the automated hyperpolarization process (*n* = 5). The rhodium concentration is below the no observed‐adverse‐effect level (NOAEL) detailed for rats in the International Council for Harmonization of Technical Requirements for Pharmaceuticals for Human Use (ICH) guideline Q3D (R1) on elemental impurities, and both MTBE and acetone are considered “Solvents with low toxic potential” according to the ICH guideline Q3C (R8) on impurities: guideline for residual solvents, and are well below their respective NOAEL

Impurity	Concentration
Rhodium	7.16 ± 0.58 µg mL^−1^
MTBE	5.7 ± 1.2 mm
Acetone	90 ± 16.6 mm

The above‐described process is performed within a fully automated single device.

In the following, this polarizer was compared to a standard d‐DNP polarizer (HyperSense, Oxford Instruments) with respect to the polarization level and differences in T_1_ and T_2_ for [1‐^13^C]pyruvate (**Table** **2**). High polarization values at the time of injection are desirable to maximize the obtainable signal. In addition, long T_1_ values before injection preserve that signal by reducing the inherent signal loss of the hyperpolarized signal caused by longitudinal relaxation. Long T_2_ values allow for repeated sampling and decreased signal loss in refocused multi‐echo experiments. We obtained slightly higher mean polarization values for DNP (22.8%) versus PHIP (17.8%) at the time of injection. Pyruvate polarized with PHIP shows a longer T_1_ and shorter T_2_ compared to d‐DNP in vitro, where the shortening of T_1_ for d‐DNP is most likely caused by the paramagnetic impurities of the d‐DNP formulation. Given that T_2_ of [1‐^13^C]pyruvate is highly pH dependent, especially in the region of 6.5 – 7.5^[^ ^28^ ^]^ and, in addition, the solvent composition strongly affects its T_2_ values^[^ ^28^ ^]^ we also expect T_2_ differences for the PHIP and d‐DNP preparations. Additionally, an increase in T_1_ by using a deuterated solvent (110.9 s) versus non deuterated solvent (56.8 s) was shown for d‐DNP. The detailed measurement procedure and data analysis can be found in the methods part of the Supporting Information. Note that these measurements are done to benchmark the hyperpolarized solutions produced by the two techniques. After intravenous injection in an animal, dilution of the hyperpolarized solution in the bloodstream removes those differences.

**Table 2:** Comparison of T_1_, T_2_ and polarization levels for d‐DNP and PHIP protocols with standard deviations given

	PHIP [D_2_O]	d‐DNP [D_2_O]	d‐DNP [H_2_O]
Polarization level [%]	17.8 ± 1.3 (*n* = 4)	22.8 ± 5.4 (*n* = 7)	–
T_1_ (1T) [s]	139.1 ± 6.1 (*n* = 7)	110.9 ± 8.7 (*n* = 6)	56.8 ± 3.1 (*n* = 5)
T_1_ (7T) [s]	105.1 ± 3.3 (*n* = 3)	92.9 ± 9.0 (*n* = 6)	61.356.7 ± 1.7 (*n* = 5)
T_2_ (7T) [s]	39.3 ± 1.8 (*n* = 3)	56.7 ± 8.8 (*n* = 6)	35.2 ± 4.1 (*n* = 5)

### 2.2 In Vivo Experiments

**Figure 2:**
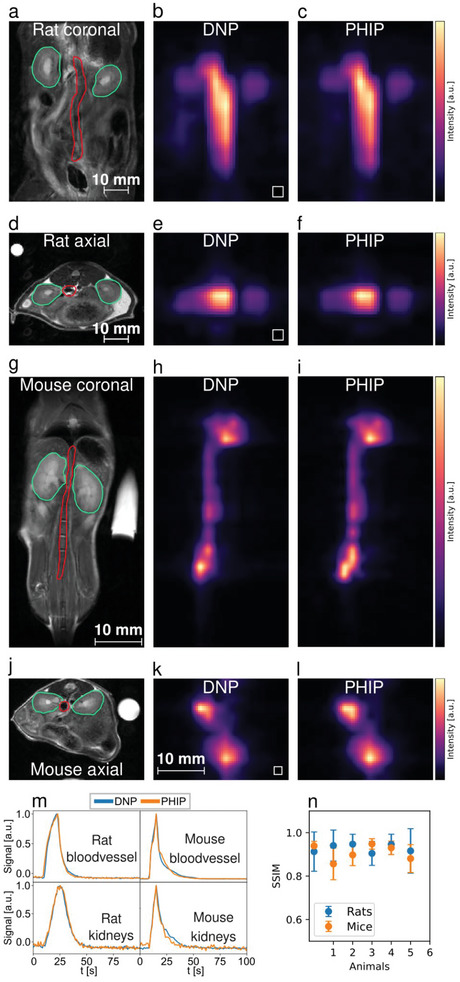
Comparison of perfusion between PHIP and d‐DNP hyperpolarized [1‐^13^C]pyruvate using a single metabolite targeted spectrally‐selective bSSFP sequence. a–f), datasets from a healthy rat where a) and d) are the anatomical reference images with the segmented regions, namely the kidneys (green) and a central blood vessel (red), b) and c) show interpolated bSSFP maximum intensity projections (MIPs) in horizontal orientation with the original resolution shown as white boxes on the lower left, e) and f) shows MIPs in axial orientation. g–l), pyruvate perfusion MIPs and their anatomical references from a healthy mouse. Visible are a central blood vessel and the heart. The signal intensities b,c,e,f,h,i,m,k,l) were scaled to maximum value for better comparability. m), signal intensity time curves from blood vessels (red ROIs) and kidneys (green ROIs), n), structural similarity indices (SSIM) showing a similar SSIM for rat (0.95 ± 0.03) and mouse (0.88 ± 0.05) measurements.

To evaluate the perfusion properties of pyruvate for both methods 12 healthy animals (6 mice, 6 rats) received two intravenous pyruvate injections with a time interval between the two injections of 30 min (PHIP and d‐DNP in mixed order). No significant difference in pyruvate‐to‐lactate conversion between first and second injection was found (Wilcoxon matched‐pairs rank test, *p* > 0.05, see Figure S4, Supporting Information). Using a frequency‐selective 3D balanced‐steady state procession (bSSFP) sequence^[^ ^29^ ^]^ pyruvate perfusion was monitored in abdominal organs and vessels over the time course of ≈100 s. Maximum intensity projections are shown in **Figure** **2** for a rat (Figure 2a–f) and a mouse (Figure 2g–l) as well as the respective time courses for the same animals (Figure 2m). For PHIP and DNP images the calculated structural similarity index showed high similarity (SSIM = 0.95 ± 0.03 and SSIM = 0.88 ± 0.05 for rats and mice, respectively).

**Figure 3:**
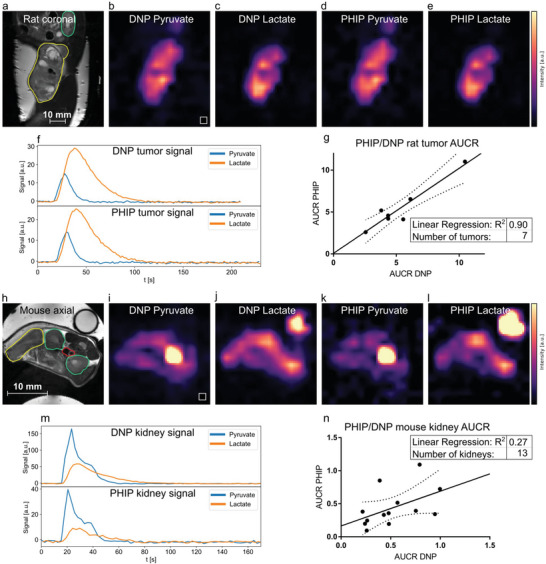
Comparison of metabolism in rat tumors and mice kidneys between PHIP and d‐DNP hyperpolarized [1‐^13^C]pyruvate using a dual metabolite targeted spectrally‐selective bSSFP sequence. a–f), PHIP and d‐DNP datasets of a Mat B III tumor‐bearing rat showing the distribution of injected hyperpolarized pyruvate b,d) and metabolized lactate c,e). In the corresponding anatomical reference (a), the tumor and kidney are drawn in yellow and green, respectively. The tumor 3D ROI time curves of pyruvate and lactate are shown in (f). The signal intensities b,c,d,e,i,j,k,l) were scaled to maximum value for better comparability. g), comparison of rat tumor AUCR of PHIP and d‐DNP showing very good correlation between the two methods (R^2^ = 0.90). h–l), pyruvate and lactate distributions in a tumor‐bearing mouse are shown, with the corresponding anatomical reference in (h). Tumor (yellow), kidneys (green), and the blood vessel (red) ROIs are depicted. A [1‐^13^C]lactate enriched phantom for RF power calibration is visible in the top right in the panels (h,j,l). m), signal intensity time curves from the left kidney (green ROI). n), correlation of PHIP and d‐DNP AUCRs of mouse kidneys (R^2^ = 0.27). Dashed lines in (g, n) indicate the 95% confidence interval.

To evaluate the metabolic conversion of pyruvate to lactate, 8 tumor‐bearing rats (Mat B III mammary adenocarcinoma) and 8 mice (EL4 lymphoma) received two intravenous pyruvate injections each with a time interval of 30 min between the two injections (PHIP and d‐DNP in mixed order). Using a frequency‐selective 3D bSSFP sequence^[^ ^29^ ^]^ alternating between pyruvate and lactate excitation, metabolism was monitored in the tumors and abdominal organs. Mean pyruvate and lactate distributions in one coronal slice for both PHIP and d‐DNP are shown exemplary for a rat tumor (**Figure** **3**,a–e) and a mouse kidney (Figure 3,h–l), together with their respective metabolite time curves (Figure 3,f,m). The calculated correlations of the area‐under‐the‐curve ratios (AUCRs)^[^ ^30^ ^]^ between PHIP and d‐DNP were found to be high for rat tumors (R^2^ = 0.90, *n* = 7) and mouse tumors (R^2^ = 0.98, see Figure S2, *n* = 3, Supporting Information) and low for mouse kidneys (R^2^ = 0.27, *n* = 13) and rat kidneys (R^2^ = 0.52, *n* = 13, see Figure S2, Supporting Information ). The kidneys’ lower R^2^ may partly be caused by the comparably lower SNR. This is due to the distances between the regions of interest and the receive surface coils in the case of rats and mice (Figure S5, Supporting Information). In addition, the mice showed generally lower perfusion of pyruvate in the kidneys than the rats, rendering the mouse kidney region‐of‐interest (ROI) signals influenced by the pyruvate of the nearby aortic blood vessel.

**Figure 4:**
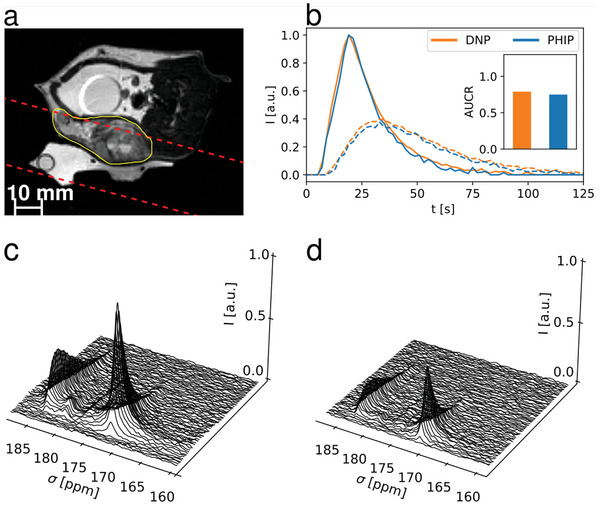
Comparison of slice‐selective spectroscopy between PHIP and d‐DNP. a), Anatomical image with slice position (red dashed) and tumor (yellow) highlighted. b) pyruvate and lactate signal time‐curves for d‐DNP and PHIP injections in a subcutaneous Mat B III rat tumor model, with area‐under‐the‐curve‐ratio (AUCR) values in the insert (d‐DNP = 0.79, PHIP = 0.75). Time curves were normalized and shifted to align their respective pyruvate peaks to facilitate direct comparison of peak shapes. Spectra shown in c) and d) had 15 Hz line broadening applied and are normalized to the largest pyruvate peak in the d‐DNP spectrum (c), showing a higher signal for the d‐DNP injection in (c), which can be explained due to a higher polarization level of the d‐DNP polarizer configuration.

**Figure 5:**
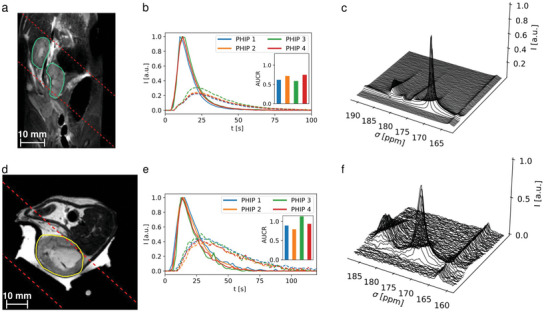
Slice‐selective spectroscopy of fast, repeated injections of PHIP polarized [1‐^13^C]pyruvate into a healthy rat a–c) and a Mat B III rat tumor model d–f). a,d) show the anatomical images with the slice position (dashed red), the kidneys (green) and the tumor (yellow) highlighted. b,e) show signal time‐curves of pyruvate and lactate, as well as the area‐under‐the‐curve ratios (AUCR) of the repeated injections as inlets, showing higher AUCR in the tumor (e, 1.06 ± 0.14) compared to a healthy kidney (b, 0.64 ± 0.05). The time curves were normalized and shifted to align their respective pyruvate peaks to facilitate direct comparison of peak shapes. c,f) show time resolved, 15 Hz line‐broadened spectra of the metabolic conversion in the tumor and kidney. A ^13^C‐urea phantom (peak at 163.5 ppm in c, f) was used for RF power calibration.

Time‐resolved slice‐selective spectroscopy was performed for both PHIP and d‐DNP in a Mat B III tumor‐bearing rat (**Figure** **4**) leading to almost qualitatively identical time curves and AUCRs (0.79 *vs*. 0.75). Furthermore, due to the short operation time of the PHIP polarizer, 4 injections were given to 2 rats (one healthy, one tumor‐bearing) with shortened time intervals of 15 minutes between injections (**Figure** **5**), successfully demonstrating a high reproducibility of the experiments AUCR = 1.06 ± 0.14, *n* = 4 (healthy animal, slice over kidneys, Figure 5b) and AUCR = 0.63 ± 0.05, *n* = 4 (tumor‐bearing animal, Figure 5e) and high experimental throughput. Comparison of the effective in vivo T_1_ values of pyruvate and lactate show similar results for PHIP and d‐DNP and good repeatability in case of the 4 repeated injections (Table S2, Supporting Information).

For all animal experiments, rectal temperature, breathing rate and oxygen saturation were logged (Figure S3, Supporting Information). However, no substantial difference between the reaction to the PHIP or d‐DNP injections on those vital parameters was found. Furthermore, there was no substantial deviation from what would be expected as a reaction due to any arbitrary intravenous injection.

## 3 Significance and Conclusion

The achievement of reproducible ≈18% polarization of purified [1‐^13^C]pyruvate at the time of injection into the animal required several key advancements in the PHIP‐SAH process. First, the precursor ester was specifically designed for more efficient hydrogenation (full hydrogenation within 5 s) and high hydrophobicity of the side‐arm residue following hydrolysis, leading to efficient washing of the residue with MTBE. In addition, the full deuteration of the CH_2_ group led to an enhancement of the singlet state order on the parahydrogenated protons. A challenge that had to be overcome was the lack of intermediate protons in the polarization transfer to the ^13^C spin.^[^ ^27^ ^]^ Careful characterization of the *J*‐coupling network showed a direct coupling of 0.4 Hz between the parahydrogenated proton and the carbon spin, sufficient for direct transfer, leading to > 36% ^13^C spin polarization. Second, the entire purification process had to be optimized and fully automated to preserve as much of the polarization as possible while reducing all impurities to acceptable levels (Table 1). The development of precursor esters of other carboxylate probes for hyperpolarized ^13^C‐MRI utilizing the same side‐arm form seems feasible.

To verify the reliability of our method, a rigorous in vivo analysis with 48 mice and rats was conducted. These experiments demonstrated that the described PHIP polarization and purification process is safe and reliable and provides the necessary signal‐to‐noise ratio for 3D metabolic imaging to obtain quantitative AUCR. We demonstrate for the hyperpolarized pyruvate solutions, independent whether produced by the PHIP or d‐DNP method, similar perfusion in healthy animals’ abdominal organs and similar pyruvate‐lactate conversion, as well as comparable effective T_1_ values (Table S2, Supporting Information). Following these results, PHIP‐based hyperpolarization seems now all set to become a widespread method for hyperpolarized MRI that has shown clear value in multiple clinical studies, including risk stratification of patients with prostate cancer,^[^ ^7^ ^]^ early treatment response monitoring of neoadjuvant chemotherapy in breast cancer patients,^[^ ^14^ ^]^ or characterization of tumor heterogeneity in glioblastoma.^[^ ^31^ ^]^ The short duration of the automated PHIP process allows for new kinds of experiments, such as leveraging rapid multi‐dose experiments to obtain new metabolic insights or access to previously unfeasible experimental designs due to long polarization durations.

In conclusion, our study demonstrates that, for the first time, PHIP‐based hyperpolarization of pyruvate can achieve comparable results with d‐DNP regarding polarization, volume and concentration levels at the time of injection, yielding quantitatively similar perfusion and metabolic information in the in vivo experiments. The polarization time for [1‐^13^C]pyruvate (≈85 s) for the PHIP prototype is much shorter than that (≈45 min) for the d‐DNP system.

## Author Contributions

L.N., M.Gi., and W.G. contributed equally to this work. L.N., M.Gi., W.G., Z.A., A.W., M.Gr., C.A.M. performed data curation; F.S., I.S. performed funding acquisition; L.N., M.Gi., W.G., Z.A., M.Gr., C.A.M., G.J.T., F.H., S.K., F.S., I.S. performed formal analysis; L.N., M.Gi., W.G., Z.A., M.Gr., C.A.M., G.J.T., F.H., S.K., F.S. performed investigation; L.N., M.Gi., W.G., Z.A., F.S., I.S. wrote the original draft; all authors discussed the results; reviewed and edited the final manuscript.

## Conflict of Interest

The authors declare the following competing financial interests: M.Gi., Z.A., P.W., F.J., S.K., C.A.M., S.L., J.S., C.M., J.B., J.H., C.V., S.K., M.K., I.S. are or were employed by NVision Imaging Technologies GmbH. F.S. serves on the scientific advisory board of NVision Imaging Technologies GmbH.

## Supporting information



Supporting Information

## Data Availability

The data that support the findings of this study are available from the corresponding author upon reasonable request.
