# Supplementary material for: Parahydrogen‐Polarized [1‐13C]Pyruvate for Reliable and Fast Preclinical Metabolic Magnetic Resonance Imaging
Source: Adv Sci (Weinh). 2023 Aug 16;10(30):2303441. doi: 10.1002/advs.202303441 (PMC10602543; doi:10.1002/advs.202303441)
Supplement: Supplementary file 1 — Supporting Information [file ADVS-10-2303441-s001.pdf]

ADVANCED SCIENCE
Open Access

Supporting Information

for *Adv. Sci.*, DOI 10.1002/advs.202303441

Parahydrogen-Polarized [1-^{13}C]Pyruvate for Reliable and Fast Preclinical Metabolic Magnetic Resonance Imaging

Luca Nagel, Martin Gierse, Wolfgang Gottwald, Zumrud Ahmadova, Martin Grashei, Pascal Wolff, Felix Josten, Senay Karaali, Christoph A. Müller, Sebastian Lucas, Jochen Scheuer, Christoph Müller, John Blanchard, Geoffrey J. Topping, Andre Wendlinger, Nadine Setzer, Sandra Sühnel, Jonas Handwerker, Christophoros Vassiliou, Frits H.A. van Heijster, Stephan Knecht, Michael Keim*, Franz Schilling* and Ilai Schwartz**

Supporting Information
©Wiley-VCH 2023
69451 Weinheim, Germany

Parahydrogen-Polarized [1-^{13}C]Pyruvate For Reliable and Fast Preclinical Metabolic Magnetic Resonance Imaging

Luca Nagel, Martin Gierse, Wolfgang Gottwald, Zumrud Ahmadova, Martin Grashei, Pascal Wolf, Felix Josten, Senay Karaali, Christoph A. Müller, Sebastian Lucas, Jochen Scheuer, Christoph Müller, John Blanchard, Geoffrey J. Topping, Nadine Setzer, Sandra Sühnel, Jonas Handwerker, Christophoros Vassiliou, Frits H.A. van Heijster, Stephan Knecht, Michael Keim, Franz Schilling, Ilai Schwartz

Abstract: Hyperpolarization techniques increase nuclear spin polarization by more than four orders of magnitude, enabling metabolic MRI. Even though hyperpolarization has shown clear value in clinical studies, the complexity, cost and slowness of current equipment limits its widespread use. Here, we demonstrate a polarization procedure of [1-^{13}C]pyruvate based on parahydrogen-induced polarization by side-arm hydrogenation (PHIP-SAH) in an automated polarizer. It was benchmarked in a study with 48 animals against a commercial dissolution dynamic nuclear polarization (DNP) device. We obtained purified, concentrated (≈ 70-160 mM) and highly hyperpolarized (≈ 18 %) solutions of pyruvate at physiological pH for volumes up to 2 ml within 85 seconds in an automated process. The safety profile, image quality, as well as the quantitative perfusion and pyruvate-to-lactate ratios, were equivalent for PHIP and DNP, rendering PHIP a viable alternative to established hyperpolarization techniques.

Table of Contents

Experimental Procedures 2
Results and Discussion 16
References 23
Author Contributions 23

Experimental Procedures

Parahydrogen induced polarization and purification of [1-^{13}C]pyruvate

The starting solution contains 220 mM [1-^{13}C]pyruvate ester, 1 mol% commercially available Rh(I)-catalyst ((dppb)(COD)$RhBF_4$), dissolved in 5.0 mL acetone-d_6. The hydrogenation reaction takes place in a heated reactor (60 °C) for eight seconds under a hydrogen pressure of 10 bar and a hydrogen flow rate of 2 standard L/min. The 90 % para-enriched hydrogen was created in a commercially available parahydrogen generator (based on DE204AF from Advanced Resource Systems, Inc.). To transfer the hydrogen polarization to the [1-^{13}C]pyruvate nuclei, the solution is shuttled into a magnetic shield (MS2, Twinleaf LLC, Princeton, US) and a radio-frequency sweep is applied. The concept of the radio-frequency sweep was recently introduced by Marshall et al.[1]. The hydrogenated solution is mixed with 1.7 ml NaOH in D_2O (400 mM, heated to 60 °C) to initiate the cleavage. Two seconds after the addition of the base, 1.7 ml of a phosphate buffer (25 mM phosphate, 2:1 mixture of NaH_2PO_4/Na_2HPO_4) is added to the solution mixture. Thereafter, 15 ml MTBE are used in each washing step. The evaporation of the residual solvents takes place in a vessel, which is heated to 50°C. While the solution is inside the vessel, nitrogen is bubbled through it at 100 mbar for 10 s. The final concentration of the injected solution was determined by dilution. For all the experiments we used 80 mM of pyruvate concentration, except for the quadruple injections in rats: Here we used 160 mM with half the volume instead, to minimize the total injected volume.

PHIP sample analysis / impurities

The purified injectables were tested on residual Rhodium with a Perkin Elmer Avio Max 220 ICP OES spectrometer. The samples were diluted in 3 % HNO_3 and referenced against a TraceCERT Yttrium standard (10 g/l in nitric acid by Merck) as an internal standard. A five point calibration was performed using a TraceCERT Rhodium standard (1 g/l in hydrochloric acid by Merck). The limit of quantification was determined at around 20 μg/l for the matrix investigated. Acetone and MTBE residues were quantified via NMR spectroscopy (Bruker Avance Neo 400 MHz).

Dissolution DNP of [1-^{13}C]pyruvate

18-62 mg 14 M [1-^{13}C]pyruvate (Merck, Darmstadt, Germany) were polarized in a commercial dissolution DNP polarizer (HyperSense, Oxford Instruments, Abingdon, UK) at 3.35 T and 1.2 K (94.133 GHz, 1.2 K, > 45 min) together with 15 mM OX063 trityl radical (GE Healthcare) and 1 mM gadoteric acid (Dotarem, Guerbet, France) and rapidly dissolved in a phosphate buffer solution (19 mM phosphate buffer, 0.1 g/L Na_2EDTA, pH adjusted to 12.8) in H_2O or D_2O, to neutralize pH and achieve an average pyruvate concentration of 80 mM. The amount of polarized [1-^{13}C]pyruvate varied because of combined polarization, T_1 and T_2 experiments such that different amounts of dissolved agent were needed.

T_1 and T_2 measurements

Measurements of T_1 at 1T were conducted using a Magritek Spinsolve NMR spectrometer. 200-450 µl of hyperpolarized [1-^{13}C]pyruvate was injected into a 5 mm NMR tube shortly after dissolution. Repeated low flip angle excitation (5 s repetition time, 20 kHz bandwidth, 40 repetitions, 4° flip angle) lead to exponential decay curves, from which T_1 was determined. Directly afterwards, 1 ml of the same sample was pipetted into a 10 mm NMR tube, placed inside a ^{13}C solenoid coil (RAPID Biomedical, Rimpar, Germany) and high field data acquired using a preclinical 7T MRI scanner (Bruker/Agilent). The pH of the solutions was measured using a pH-electrode and resulted in the following values: DNP (D2O) = 7.27 ± 0.38 (n = 6), DNP (H2O) = 7.45 ± 0.37 (n = 5), PHIP (1T, D2O) = 6.72 ± 0.26 (n = 7), and PHIP (7T, D2O) = 7.22 ± 0.20 (n=3). Variations in pH for DNP measurements could be due to adjustments of the buffer solution during the experiments.

T_1 and T_2 values were measured using a repeated excitation non-selective spectroscopy sequence (NSPECT, TR 5 s, 40 repetitions, 2° flip angle block pulse with 10 kHz transmit bandwidth, acquisition duration 340 ms, receiver bandwidth 6 kHz, 2048 acquired points per excitation) followed by a Carr-Purcell-Meiboom-Gill (CPMG) sequence (TR 90 s, echo spacing 10 ms, 8192 echoes per excitation, 90° excitation and 180° refocusing block pulses with 5 kHZ transmit bandwidth, receiver bandwidth 5 kHz, 32 acquired points per echo, acquisition duration 6.4 ms, 1 ms spoilers at 20% strength around each refocusing pulse)[2]. For T_1 measurements, the spectra were integrated and then fitted to a mono-exponential decay function. Similarly for T_2 measurements, the decay curve resulting from the CPMG measurement was fitted resulting in the values shown in Table

1. Data processing was done using Python 3.10.10 self-written scripts in combination with Jupyter Notebooks with loading routine functions from[3].

Polarization level determination

Polarization levels were determined according to:

$$P_{lvl,hyp} = P_{lvl,th} * \frac{S_{hyp} sin(\alpha_{th})}{S_{th} sin(\alpha_{hyp})} * \sqrt{N_{thermal}} ,$$

where the thermal polarization level is calculated by:

$$P_{lvl,th} = tanh(\frac{\hbar\omega B}{2kT}),$$

with $\hbar$ = 1.05*10^{-34} J·s, ω = 67.3*10^{6} rad·s^{-1}T^{-1} , k = 1.38*10^{-23} J·K^{-1} and T = 301.6 K. The measurement was done by comparing the first spectrum of a low flip angle full volume excitation measurement (TR = 5 s, α_{hyp} = 4°, 40 repetitions) to a thermal measurement of the same sample with longer repetition time (TR > 5*T_1 (560-1050 s),α_{th} = 90°, $N_{thermal}$ = 73-103)[4]. Both hyperpolarized and thermally polarized real spectra were phase- and baseline-corrected. The spectra were normalized by dividing by the standard-deviations of the noise in a background region of the spectra to enable SNR comparison between hyperpolarized and thermal spectra. The spectra were then integrated in a region of 10 ppm around the [1-^{13}C]pyruvate peak (S_{hyp}). Thermal spectra were corrected similarly, averaged and integrated (S_{th}). Results were corrected for differences in flip angle and number of thermal averages.

Data processing was done using Python 3.10.10 self-written scripts in combination with Jupyter Notebooks with loading routine functions from[3].

Animal experiments

Animals experiments were conducted according to relevant laws and regulations and were approved by an ethical review board (Regierung von Oberbayern, ROB-55.2-2532.Vet_02-17-177 and related amendments). MR Imaging of healthy subjects for assessment of perfusion was performed in twelve female healthy C57BL/6 mice (m = 17.2 ± 0.5 g, 6-7 weeks old, Charles River) and twelve female healthy Wistar rats (m = 192.2 ± 12.2 g, 6-7 weeks old, Charles River). MR Imaging of real-time metabolic conversion was performed in subcutaneous tumor-bearing animals. Here, twelve female C57BL/6 mice (m = 19.6 ± 1.2 g, 7-8 weeks old, Charles River) injected with 5 x 10^6 EL4 lymphoma cells, suspended in 100 µL PBS, in the right lower flank and twelve female Wistar rats (m = 212.2 ± 11.0 g, 7-8 weeks old, Charles River) injected with 2 x 10^6 Mat B III adenocarcinoma cells, suspended in 100 µL PBS, in the right lower flank. Tumors were investigated when grown to 10 mm for mice and 28 mm for rats, occurring typically on days 6-10 and days 13-17 for mice and rats respectively. Anesthesia for imaging was initiated with 5 % isoflurane in 100 % oxygen at a flow rate of 2 l/min and maintained at an isoflurane level of 1 - 3 %. Tail vein catheters were inserted prior to animal positioning. For hyperpolarized MRI acquisitions, mice or rats were injected with 240 µL or 1200 µL (450 µL in case of the 4 times repeated injection into a rat), respectively, of dissolved hyperpolarized agent (either DNP or PHIP) 28.9 ± 7.0 s and 24.8 ± 3.4 s post agent preparation completion respectively. Injection times for PHIP- and DNP-prepared agents were identical for mice (PHIP: 7.9 ± 2.1 s; DNP: 8.0 ± 2.1 s) and for rats (PHIP: 15.3 ± 2.9 s; DNP: 15.5 ± 4.3 s).

Animal experiments:

Date	Animal	Animal No.	Health	DNP/PHIP	sequence
2023.01.30	Rat	1	healthy	DNP	bssfp perfusion
2023.01.30	Rat	1	healthy	PHIP	bssfp perfusion
2023.01.30	Rat	2	healthy	PHIP	bssfp perfusion
2023.01.30	Rat	2	healthy	DNP	bssfp perfusion
2023.01.30	Rat	3	healthy	DNP	bssfp perfusion
2023.01.30	Rat	3	healthy	PHIP	bssfp perfusion
2023.01.31	Mouse	4	healthy	DNP	bssfp perfusion
2023.01.31	Mouse	5	healthy	PHIP	bssfp perfusion
2023.01.31	Mouse	5	healthy	DNP	bssfp perfusion
2023.01.31	Mouse	6	healthy	DNP	bssfp perfusion
2023.01.31	Mouse	6	healthy	PHIP	bssfp perfusion
2023.02.01	Rat	7	healthy	PHIP	bssfp perfusion
2023.02.01	Rat	7	healthy	DNP	bssfp perfusion
2023.02.01	Rat	8	healthy	DNP	bssfp perfusion
2023.02.01	Rat	8	healthy	PHIP	bssfp perfusion
2023.02.01	Rat	9	healthy	PHIP	bssfp perfusion
2023.02.01	Rat	9	healthy	DNP	bssfp perfusion
2023.02.02	Mouse	10	healthy	DNP	bssfp perfusion
2023.02.02	Mouse	10	healthy	PHIP	bssfp perfusion
2023.02.02	Mouse	11	healthy	PHIP	bssfp perfusion
2023.02.02	Mouse	11	healthy	DNP	bssfp perfusion
2023.02.02	Mouse	12	healthy	DNP	bssfp perfusion
2023.02.02	Mouse	12	healthy	PHIP	bssfp perfusion
2023.02.03	Mouse	5	healthy	DNP	bssfp perfusion
2023.02.03	Mouse	5	healthy	PHIP	bssfp perfusion
2023.02.03	Mouse	6	healthy	PHIP	bssfp perfusion
2023.02.03	Mouse	6	healthy	DNP	bssfp perfusion
2023.02.03	Rat	7	healthy	DNP	bssfp perfusion
2023.02.03	Rat	7	healthy	PHIP	bssfp perfusion
2023.02.06	Rat	13	MAT B III	DNP	bssfp metabolism
2023.02.06	Rat	13	MAT B III	PHIP	bssfp metabolism

2023.02.06	Rat	14	MAT B III	PHIP	bssfp metabolism
2023.02.06	Rat	14	MAT B III	DNP	bssfp metabolism
2023.02.07	Rat	15	MAT B III	PHIP	bssfp metabolism
2023.02.07	Rat	15	MAT B III	DNP	bssfp metabolism
2023.02.07	Rat	16	MAT B III	PHIP	bssfp metabolism
2023.02.07	Rat	16	MAT B III	DNP	bssfp metabolism
2023.02.07	Mouse	17	EL IV	PHIP	bssfp metabolism
2023.02.07	Mouse	17	EL IV	DNP	bssfp metabolism
2023.02.08	Mouse	18	EL IV	DNP	bssfp metabolism
2023.02.08	Mouse	18	EL IV	PHIP	bssfp metabolism
2023.02.08	Mouse	19	EL IV	DNP	bssfp metabolism
2023.02.08	Mouse	19	EL IV	PHIP	bssfp metabolism
2023.02.08	Mouse	20	EL IV	PHIP	bssfp metabolism
2023.02.08	Mouse	20	EL IV	DNP	bssfp metabolism
2023.02.09	Rat	21	MAT B III	DNP	bssfp metabolism
2023.02.09	Rat	21	MAT B III	PHIP	bssfp metabolism
2023.02.09	Mouse	22	EL IV	DNP	bssfp metabolism
2023.02.09	Mouse	22	EL IV	PHIP	bssfp metabolism
2023.02.09	Mouse	23	EL IV	PHIP	bssfp metabolism
2023.02.09	Mouse	23	EL IV	DNP	bssfp metabolism
2023.02.10	Rat	24	MAT B III	DNP	bssfp metabolism
2023.02.10	Rat	24	MAT B III	PHIP	bssfp metabolism
2023.02.13	Rat	25	MAT B III	DNP H_2O	bssfp metabolism
2023.02.13	Rat	25	MAT B III	DNP D_2O	CSI
2023.02.13	Rat	26	MAT B III	DNP H_2O	bssfp metabolism
2023.02.13	Rat	26	MAT B III	DNP D_2O	bssfp metabolism
2023.02.14	Rat	27	MAT B III	DNP	bssfp metabolism
2023.02.14	Rat	27	MAT B III	PHIP	bssfp metabolism
2023.02.14	Rat	28	MAT B III	DNP	bssfp metabolism
2023.02.14	Rat	28	MAT B III	PHIP	bssfp metabolism
2023.02.15	Rat	29	MAT B III	PHIP	Slice selective spectroscopy
2023.02.15	Rat	29	MAT B III	DNP	Slice selective spectroscopy

2023.02.15	Rat	30	MAT B III	4x PHIP	Slice selective spectroscopy
2023.02.15	Mouse	31	EL IV	PHIP	bssfp metabolism
2023.02.15	Mouse	31	EL IV	DNP	bssfp metabolism
2023.02.16	Mouse	32	EL IV	PHIP	bssfp metabolism
2023.02.16	Mouse	32	EL IV	DNP	bssfp metabolism
2023.02.16	Mouse	33	EL IV	DNP	Slice selective spectroscopy
2023.02.16	Mouse	33	EL IV	PHIP	Slice selective spectroscopy
2023.02.16	Mouse	34	EL IV	PHIP	Slice selective spectroscopy
2023.02.16	Mouse	34	EL IV	DNP	Slice selective spectroscopy
2023.02.17	Mouse	35	EL IV	DNP	CSI
2023.02.17	Mouse	35	EL IV	PHIP	CSI
2023.02.17	Mouse	36	EL IV	PHIP	CSI
2023.02.17	Mouse	36	EL IV	DNP	CSI
2023.03.23	Mouse	37	healthy	DNP	bssfp metabolism
2023.03.23	Mouse	38	healthy	PHIP	bssfp metabolism
2023.03.23	Mouse	39	healthy	DNP	bssfp metabolism
2023.03.23	Mouse	39	healthy	PHIP	bssfp metabolism
2023.03.23	Mouse	40	healthy	PHIP	CSI
2023.03.23	Mouse	40	healthy	PHIP	CSI
2023.03.24	Mouse	41	healthy	PHIP	bssfp metabolism
2023.03.24	Mouse	41	healthy	DNP	bssfp metabolism
2023.03.24	Mouse	42	healthy	PHIP	bssfp metabolism
2023.03.24	Mouse	42	healthy	PHIP	bssfp metabolism
2023.03.27	Rat	43	MAT B III	4x PHIP	Slice selective spectroscopy
2023.03.27	Rat	44	MAT B III	PHIP	bssfp metabolism
2023.03.27	Rat	44	MAT B III	DNP	bssfp metabolism
2023.03.29	Rat	45	MAT B III	PHIP	bssfp metabolism
2023.03.29	Rat	45	MAT B III	DNP	bssfp metabolism
2023.03.29	Rat	46	MAT B III	PHIP	bssfp metabolism
2023.03.29	Rat	46	MAT B III	PHIP	CSI

2023.03.31	Rat	47	MAT B III	DNP	bssfp metabolism
2023.03.31	Rat	47	MAT B III	PHIP	bssfp metabolism
2023.03.31	Rat	48	MAT B III	DNP	bssfp metabolism
2023.03.31	Rat	48	MAT B III	PHIP	bssfp metabolism

Table ST1: The table lists in detail how many experiments using a certain sequence were conducted. The “Health” column indicates whether the animals were healthy or tumor-bearing (EL IV in case of mice and MAT B III in case of rats). The data obtained using a CSI sequence were found to be of minor relevance to this study and therefore not shown.

Animal monitoring

Animal body temperature was kept at 37-39 °C target temperature using warm air being blown through the magnet bore with a PET Dryer Model B-8 (XPower, City of Industry, CA, USA). The body temperature was measured with a rectal MR-compatible Pt100 probe (SA Instruments, Stony Brook, NY, USA). Oxygen saturation (SpO_2) was measured only for rats using a near-infrared photometric probe (SA Instruments) placed on a hind paw. For animal condition monitoring, temperature, breathing and SpO_2 were sampled every 1 s and stored in a log file with a preclinical monitoring system Model 1030 (SA Instruments). Each log file covered the entire imaging session from animal placement to removal from the scanner. Example monitoring data for a rat and a mouse are displayed in Supporting Information Figure S3.

Scanner and Hardware

MRI animal measurements were carried out using a preclinical 7T small animal scanner (Discovery MR901 magnet and gradient system, Agilent, Santa Clara, CA, USA; AVANCE III HD electronics, Bruker, Billerica, MA, USA). For mouse experiments, a dual-tuned $^{1}H/^{13}C$ birdcage resonator (RAPID Biomedical, Rimpar, Germany) with an inner diameter of 31 mm was used.

For rat experiments, a dual-tuned $^{1}H/^{13}C$ volume resonator (inner diameter 72 mm, RAPID Biomedical) was used for proton anatomical imaging, shimming, frequency calibration, and ^{13}C excitation. Two surface receiver ^{13}C coils (20 mm diameter, RAPID Biomedical) were positioned on the tumor for slice spectroscopy experiments, a two channel flexible coil ^{13}C receive array (RAPID Biomedical, Germany) was used for the perfusion and metabolic bSSFP acquisitions To ensure enhanced B_0 shim uniformity during both mouse and rat experiments, Carbopol® 980 gel (Caesar & Loretz GmbH, Germany) was applied to surround the tumor.
A phantom containing 300 μl of 2 M [1-^{13}C]-lactate and 1 mM gadoteric acid (Dotarem, Guerbet, Villepinte, France) was positioned next to the animal for ^{13}C RF power calibration.
The pH of NMR samples was measured after spectroscopy measurements using a pH-Combination Electrode N 6000 A and a ProLab 4000 multiparameter benchtop meter (SI analytics, Mainz, Germany).

In vivo MR spectroscopy

Slice spectroscopy was performed by applying an 8° RF pulse every 2 s for 100 repetitions. The excitation used a Shinnar-Le Roux RF pulse of sharpness 3, a duration of 0.35 s, an excitation bandwidth of 12 kHz, and slice thickness 25.5 mm, and the receiver bandwidth was 2200.7 Hz, with a spectral resolution of 0.54 Hz/point. Lorentzian functions were fitted to the baseline corrected spectral data for pyruvate and lactate peaks. Subsequently these fits were integrated to obtain time curves and AUCRs.

In vivo MRI

Anatomical references were acquired using a multislice T_2-RARE sequence. Mouse imaging was done using a repetition time of 5 s, echo time 6.67 ms, RARE factor 12, number of averages 12, in-plane pixel size 0.175 x 0.175 mm^2, and slice thickness 1.75 mm. Anatomical images of rats were acquired using a repetition time of 4 s, echo time 6.33 ms, RARE factor 12, number of averages 9, in-plane pixel size 0.3 x 0.3 mm^2, and slice thickness 4.5 mm.

A spectrally-selective 3D-balanced steady-state free precession (3D-bSSFP) sequence was used to either measure perfusion of hyperpolarized [1-^{13}C]pyruvate or to image [1-^{13}C]pyruvate and [1-^{13}C]-lactate distribution in an alternating pattern[5].

Pyruvate perfusion was imaged with the sequence set to play constant-phase radiofrequency (RF) pulses with an $\alpha/2$-TR preparation pulse and repetition time 6.56 ms. The RF pulses were Shinnar-Le Roux pulses of sharpness 1, 2.565 ms duration, 819 Hz excitation bandwidth and

flipangle 6°. The pyruvate targeting RF pulse was offset by -383 Hz (~2.5 passbands) from the pyruvate resonance frequency.

Metabolic imaging was performed using the same pulse as in the perfusion experiments but alternating the offset frequency and flip angle after each 3D image acquisition between -383 Hz and 6° (pyruvate targeting) and 537 Hz (~3.5 passbands) and 40° (lactate targeting).

The spatial resolution was 1.75x1.75x1.75 mm^3 for mouse experiments and 4.5x4.5x4.5 mm^3 for rat experiments. The measurements were started before injection and 150 (mice) and 250 (rats) images were acquired.

Perfusion and calculation of structural similarity index (SSI)

Complex-valued bSSFP image stacks were reconstructed using the built-in ParaVision 7 (Bruker) reconstruction pipeline. Images were loaded using Python scripts (loading routine functions based on [6,7]) and then processed with self-written analysis scripts . Images were shifted to align with anatomical reference images. For each voxel, 20 consecutive repetitions around the pyruvate peak were summed and absolute values were taken. A background region with no signal present was chosen and its mean was subtracted from each bSSFP image stack (separately for PHIP and DNP injection experiments). Slices with visible signal were chosen by visual inspection in three orientations (axial, coronal and sagittal). For each two-dimensional bSSFP slice pair acquired from the same animal after a PHIP and DNP injection a structural similarity index (SSI) was computed using the structural-similarity function from the scikit-image Python package (version 0.19.3) with default parameters[8]. The values for the three orientations were then averaged for each animal.

Generation of 3D masks

Each 4D dataset was interpolated (nearest neighbor) to the resolution of the coronal reference images. 2D regions of interest (ROIs) were drawn onto each slice of the coronal reference and stacked together to yield 3D ROI masks.

Area under the curve ratio (AUCR)

For calculation of the AUCR of lactate to pyruvate, the four dimensional (3D spatial + 1D temporal) image datasets were shifted in space to align with the anatomical reference image. This is necessary due to the relatively small matrix size of the metabolic images. In the rat exams, the signals coming from the two receiver coil channels were additionally processed using a root-mean-square channel combination. 3D ROI masks were applied to the 4D magnitude metabolic datasets. The masked 4D ROIs were averaged along all 3 spatial dimensions, resulting in a 1D ROI signal time curve, yielding essentially a weighted average, with the weights the proportional contribution of the masked voxels to the signal. An average noise term, calculated from a signal-free time frame was subtracted from both the pyruvate and lactate time curves. The resulting time curves were summed, yielding the area under the curve (AUC) and, after division, the AUC ratio (AUCR) of the masked ROI.

Signal-to-noise ratio (SNR)

A signal-to-noise (SNR) threshold was applied to the pyruvate AUCs. The pyruvate AUC $\boldsymbol{AUC_{pyr}}$ was calculated as weighted means as described above. The pyruvate SNR $\boldsymbol{SNR_{pyr}}$ was defined as:

$$\boldsymbol{SNR_{pyr}} = \frac{\boldsymbol{AUC_{pyr} - Noise_{pyr}}}{\boldsymbol{Noise_{pyr}/N_{voxel}}}$$

With AUC_{pyr} the mean AUC in the ROI, $Noise_{pyr}$ the signal-free mean noise level and N_{voxel} the number of voxels in the ROI. ROIs with $\boldsymbol{SNR_{pyruvate}}$ < 5 were excluded from the analysis.

Data analysis and software availability

The Python code used for the MRI/MRS parts of this publication is published under https://github.com/QuE-MRT/2023_TUMKRI_NVIS_PHIPvsDNPInVivoStudy with exemplary notebooks for perfusion, metabolism, slice spectroscopy and in vitro (T_1, T_2, polarization) measurements.

Results and Discussion

Table ST2:

Experiment	T_1 [s] Pyruvate	T_1 [s] Lactate	Figure
Rat tumor DNP	17.8 ± 0.2	42.9 ± 1.0	4 b, c
Rat tumor PHIP	15.2 ± 0.2	41.3 ±1.4	4 b, d
Rat kidney 4x PHIP	10.1 ± 0.6	24.4 ± 0.3	5 b
Rat tumor 4x PHIP	13.8 ± 1.0	39.9 ± 1.0	5 e

Caption ST2: The table lists effective T_1 values of hyperpolarized [1-^{13}C]pyruvate and [1-^{13}C]lactate of the slice spectroscopy experiments in rats (Figures 4, 5). The effective T_1 values were determined by fitting a mono exponential decaying function and the effect of additional signal loss due to excitation was considered. The relatively short T_1 of hyperpolarized pyruvate could be explained by the flow suppression effect from the slice selective gradient.

Figure S1.

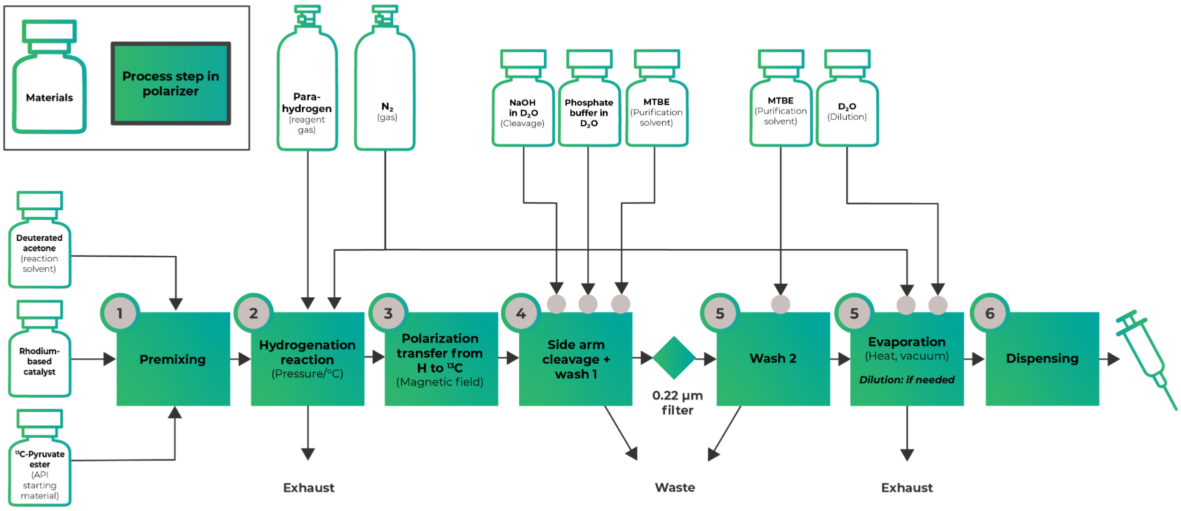

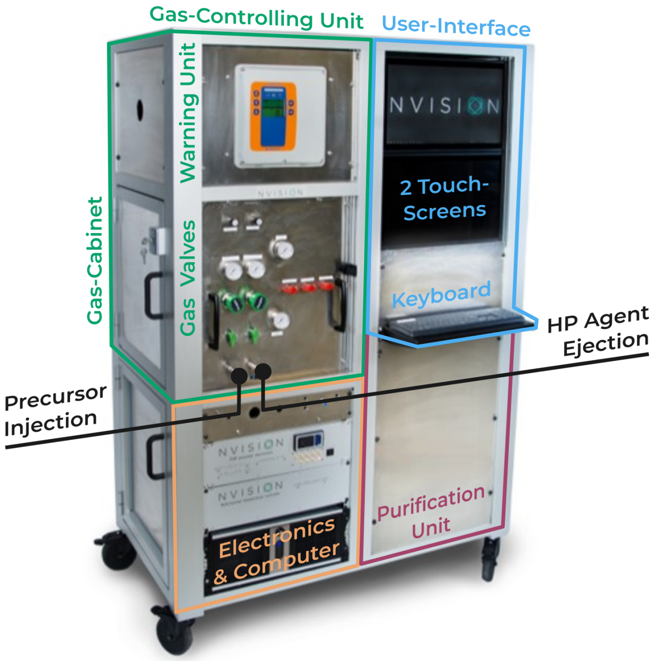

Caption Figure S1: Schematic of the automated polarization process and photograph with description of key elements of the polarizer prototype.

Figure S2

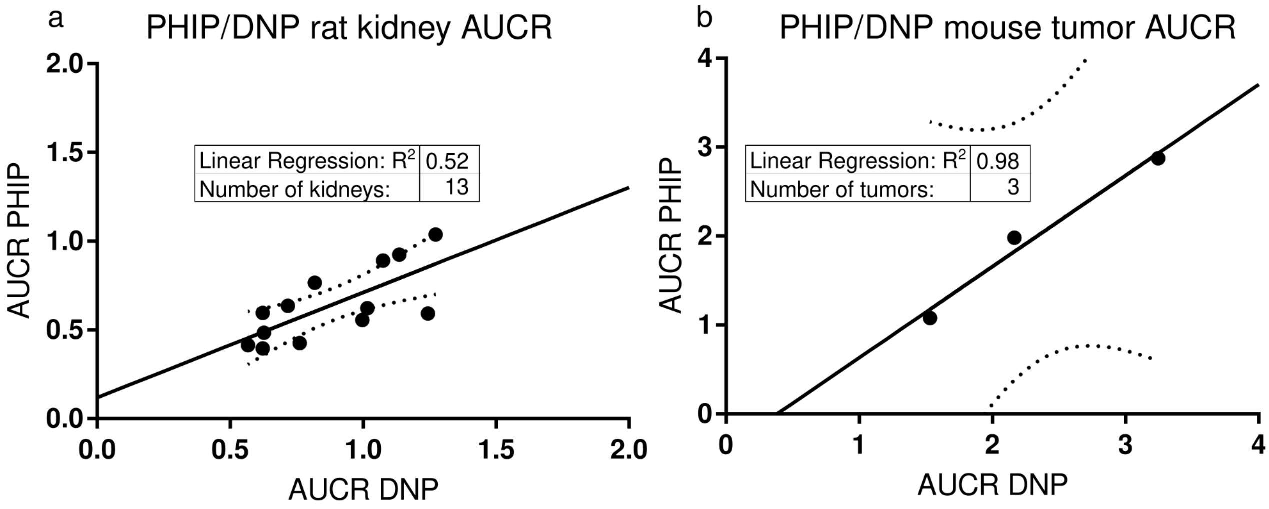

Caption Figure S2: Area under the curve ratios (AUCR) of lactate to pyruvate for rat kidneys (a) and mouse tumors (b). The AUCR values taken from rat kidney ROIs show moderate correlation ($R^2 = 0.52$). The rat kidneys were relatively far away from the two ^{13}C receive surface coils resulting in comparatively noisy data. AUCR values obtained from mouse tumor ROIs show strong relationship ($R^2 = 0.98$). However, it should be noted that only three tumor ROIs had sufficient signal-to-noise ratio (SNR) for meaningful comparison. Dashed lines indicate the 95 % confidence interval.

Figure S3.

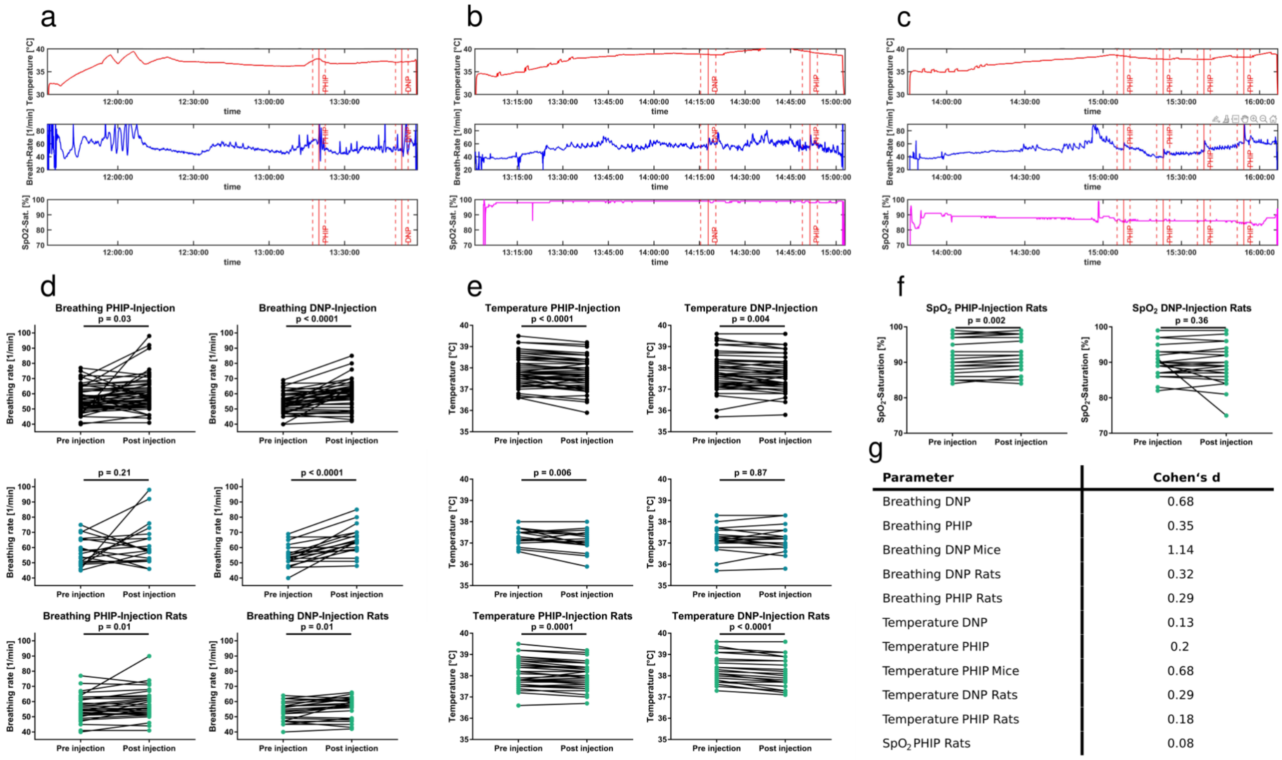

Parameter	Cohen's d
Breathing DNP	0.68
Breathing PHIP	0.35
Breathing DNP Mice	1.14
Breathing DNP Rats	0.32
Breathing PHIP Rats	0.29
Temperature DNP	0.13
Temperature PHIP	0.2
Temperature PHIP Mice	0.68
Temperature DNP Rats	0.29
Temperature PHIP Rats	0.18
SpO_2 PHIP Rats	0.08

Caption Figure S3: Rectal temperature, breathing rate and oxygen saturation were logged for mice (a, example), rats (b, example) which received one PHIP- and DNP-polarized pyruvate injection (red solid lines). (c), one animal received four PHIP-polarized private injections, spaced by 15 minutes each. To assess the influence of injections from both polarization techniques on animal condition, monitoring curves for all three parameters (breathing rate, temperature, SpO_2) were averaged 150 seconds prior and post injections (ranges indicated by dashed red lines in (a, b, c)) and data analysis was performed for mice and rats separately as well as for pooled data. Differences for pre- and post-injection parameters were assessed using paired t-tests. Here, breathing rates for both, PHIP injections and DNP-injections for all animals (d, top) show a slight increase following injection (PHIP: $p = 0.03$, $\Delta bpm = 4\ min^{-1}$; DNP: $p < 0.0001$, $\Delta bpm = 6\ min^{-1}$). While this is found for both mice (d, middle right)

and rats (d, bottom right) for DNP injections (mice: $p < 0.0001$; rats: $p = 0.01$), only rats show a temporary increase in breathing rate for PHIP injections (d, bottom left, $p = 0.01$). (e), measured rectal temperature decreases slightly for both PHIP-injections (e, top left, $p < 0.0001$, $\Delta T = -0.2°C$) and DNP injections (e, top right, $p = 0.004$, $\Delta T = -0.1°C$). Again, rats show this trend more consistently for both PHIP (e, bottom left, $p < 0.0001$) and DNP (e, bottom right, $p < 0.0001$) compared to mice (e, middle left + right). f, blood oxygen saturation levels could only be measured for rats, where PHIP injections show a slight change in measured SpO_2-level (f, left, $p = 0.002$) while DNP-injections are unobtrusive (f, right). (g), while the majority of the groups show significant effects for parameter changes upon injection, these effects are of minor or no importance regarding the tolerance of the polarized injections as assessed by Cohen's d. Apart from the breathing rate for mice during DNP-injections ($d = 1.14$), which can be explained by the substantial injection volume in relation to the total mouse blood volume, no parameter regardless of injection type shows a remarkable effect size.

Figure S4

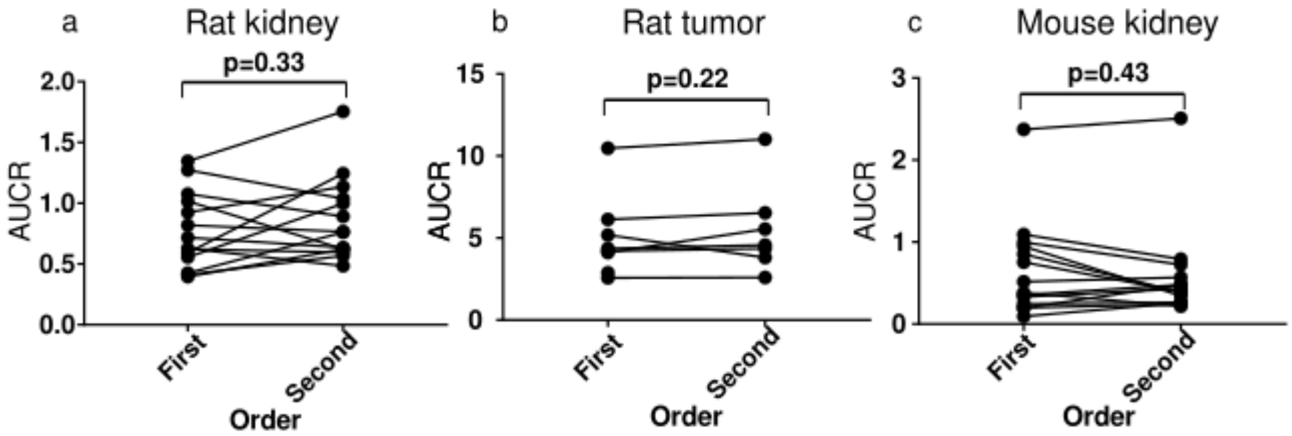

Caption Figure S4: Influence of order of injection on area under the curve ratio (AUCR). A waiting time of 30 minutes was kept in between injections of hyperpolarized [1-^{13}C]pyruvate into the same animal. The area under the curve ratio (ACUR) of lactate to pyruvate for rat kidneys (a), rat tumors (b) and mouse kidneys (c) were grouped into first and second injection. Differences in AUCR determined by first or second injection were assessed using a Wilcoxon matched-pairs rank test. The AUCR in rat kidneys shows a slight increase from first to second injection (a, +0.0063, $p = 0.33$) a well as the AUCR in rat tumors (b, +0.22, $p = 0.22$) and the AUCR in mouse kidneys (c, +0.003, $p = 0.43$). None of the found differences (a, b, c) was of statistical significance ($p > 0.05$)

Figure S5

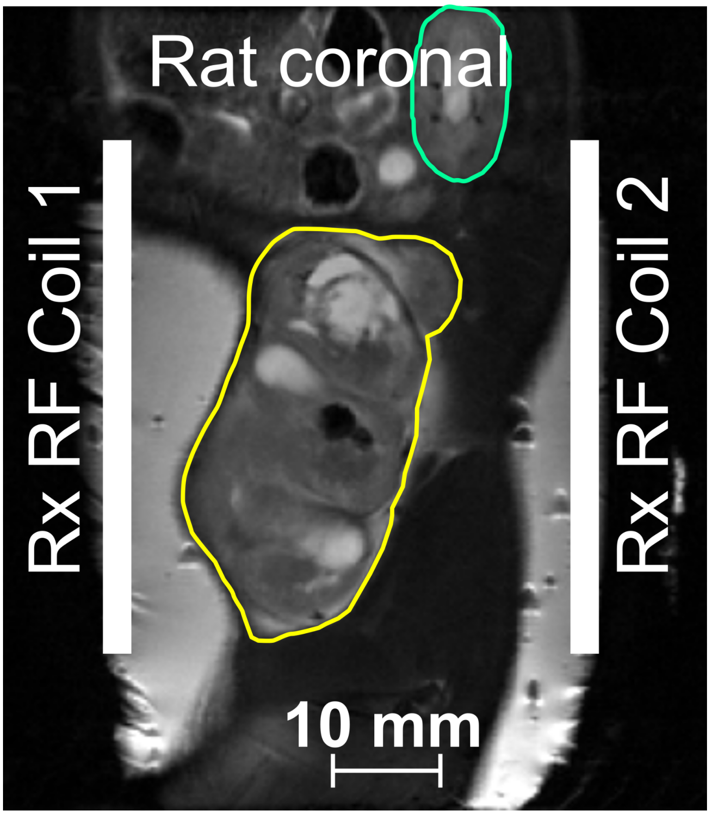

Caption Figure S5: Position of the two channel flexible coil ^{13}C receive (Rx) array for rat measurements. While the tumor (yellow) was well centered in the most sensitive region of the receive coils, the kidney (green) was only partially covered.

References

[1] A. Marshall, A. Salhov, M. Gierse, C. Müller, M. Keim, S. Lucas, A. Parker, J. Scheuer, C. Vassiliou, P. Neumann, F. Jelezko, A. Retzker, J. W. Blanchard, I. Schwartz, S. Knecht, *J. Phys. Chem. Lett.* **2023**, *14*, 2125–2132.
[2] M. Grashei, C. Hundshammer, F. H. A. van Heijster, G. J. Topping, F. Schilling, *Pharmaceuticals* **2021**, *14*, 327.
[3] Benno Meier, **2022**.
[4] A. Ferrari, J. Peters, M. Anikeeva, A. Pravdivtsev, F. Ellermann, K. Them, O. Will, E. Peschke, H. Yoshihara, O. Jansen, J.-B. Hövener, *Sci. Rep.* **2022**, *12*, 11694.
[5] J. G. Skinner, G. J. Topping, L. Nagel, I. Heid, C. Hundshammer, M. Grashei, F. H. A. Van Heijster, R. Braren, F. Schilling, *Magn. Reson. Med.* **2023**, mrm.29676.
[6] NMR group of the Institute of Scientific Instruments of the Czech Academy of Sciences, **2020**.
[7] Jörg Döpfert, **2014**.
[8] S. Van Der Walt, J. L. Schönberger, J. Nunez-Iglesias, F. Boulogne, J. D. Warner, N. Yager, E. Gouillart, T. Yu, *PeerJ* **2014**, *2*, e453.

Author Contributions

Data curation: LN, MGi, WG, ZA, AW, MGr, CAM

Funding acquisition: FS, IS

Formal analysis: LN, MGi, WG, ZA, MGr, CAM, GJT, FvH, SK, FS, IS

Investigation: LN, MGi, WG, ZA, MGr, CAM, GJT, FvH, SK, FS

Writing of original draft: LN, MGi, WG, ZA, FS, IS

Discussion of results: all authors

Reviewing and editing of manuscript: all authors
